# Human Mesenchymal Stem Cell Morphology and Migration on Microtextured Titanium

**DOI:** 10.3389/fbioe.2016.00041

**Published:** 2016-05-10

**Authors:** Brittany L. Banik, Thomas R. Riley, Christina J. Platt, Justin L. Brown

**Affiliations:** ^1^Musculoskeletal Regenerative Engineering Laboratory, Department of Biomedical Engineering, The Pennsylvania State University, University Park, PA, USA; ^2^Perelman School of Medicine, University of Pennsylvania, Philadelphia, PA, USA; ^3^Department of Electrical Engineering, The Pennsylvania State University, University Park, PA, USA

**Keywords:** cell–material interactions, titanium (alloys), PEEK, spinal implant, regenerative medicine

## Abstract

The implant used in spinal fusion procedures is an essential component to achieving successful arthrodesis. At the cellular level, the implant impacts healing and fusion through a series of steps: first, mesenchymal stem cells (MSCs) need to adhere and proliferate to cover the implant; second, the MSCs must differentiate into osteoblasts; third, the osteoid matrix produced by the osteoblasts needs to generate new bone tissue, thoroughly integrating the implant with the vertebrate above and below. Previous research has demonstrated that microtextured titanium is advantageous over smooth titanium and PEEK implants for both promoting osteogenic differentiation and integrating with host bone tissue; however, no investigation to date has examined the early morphology and migration of MSCs on these surfaces. This study details cell spreading and morphology changes over 24 h, rate and directionality of migration 6–18 h post-seeding, differentiation markers at 10 days, and the long-term morphology of MSCs at 7 days, on microtextured, acid-etched titanium (endoskeleton), smooth titanium, and smooth PEEK surfaces. The results demonstrate that in all metrics, the two titanium surfaces outperformed the PEEK surface. Furthermore, the rough acid-etched titanium surface presented the most favorable overall results, demonstrating the random migration needed to efficiently cover a surface in addition to morphologies consistent with osteoblasts and preosteoblasts.

## Introduction

Spinal fusion surgery combines (or fuses) two or more vertebrae together to reduce discomfort by immobilizing a painful vertebral motion segment and restoring spinal stability (Williams et al., 2005; Nouh, 2012; Obrigkeit et al., 2012). Following surgery, it can take 6–12 months for the fusion process to occur (Obrigkeit et al., 2012). During the fusion process, implant osseointegration is critical (Olivares-Navarrete et al., 2010).

Autografts and allografts are often viewed as the “gold standard” in many biomedical applications; however, bone material donations have complications. Specifically for spinal fusion cages, the issues include an unpredictable nature due to an inconsistency in mechanical strength, machining challenges, and migration issues (Rihn et al., 2009; Obrigkeit et al., 2012). Titanium and polyetheretherketone (PEEK) implants are among the most common alternatives to bone (Abernathie and Pfeiffer, 2011; Cabraja et al., 2012; Obrigkeit et al., 2012).

Cell–material interactions are of particular interest in biomedical implants because the initial contact between the cells and the biomaterial can define the success of the device. As part of the tissue microenvironment presented to the cells, the surface morphology and chosen material are integral in this interaction and the cellular response – adhesion, spreading, migration, proliferation, and differentiation – ultimately contributing to the fate of the cells and tissue formation (Bächle and Kohal, 2004; Anselme and Bigerelle, 2005; Zhao et al., 2011). The surface features on the fusion host environment have key roles in the fusion process.

Textured surface features are of specific interest because cells interact with the extracellular environment through micro- (e.g., organelles) and nanoscale (e.g., protein complexes, such as focal adhesions). When a surface demonstrates a characteristic dimension on the same order of magnitude as protein complexes up to organelles, the response of the cell can be modulated through a myriad of intracellular signaling and mechanotransduction events, leading to altered gene transcription and potentially regulating the differentiation of stem and progenitor cells (Anselme et al., 2010; Ozdemir et al., 2013; Higgins et al., 2015). We believe that the presentation of textured surfaces to cells is a non-toxic, material-independent option to induce beneficial cellular responses for medical devices and serve as a tool to help design more therapeutically effective biomedical implants.

This research investigates the effects of spinal fusion cage surface morphology on initial cellular responses. Adhesion, spreading, migration, proliferation, and differentiation are important phenotypic considerations. This article directly addresses spreading and migration through morphology, speed of movement, differentiation markers, and directionality data, and indirectly suggests potential differentiation outcomes through circularity and cell spreading (*via* aspect ratio) measurements. Surface characteristics influence numerous fields, including proliferation, gene expression activity, phenotype commitment, cell adherence, protein adsorption, and cell shape (Deligianni et al., 2001; Olivares-Navarrete et al., 2010). These cell–material interactions are significant toward discerning the potential for bacterial growth on the implant and in turn suggest the chance for a biomaterial-associated infection (BAI).

The term “race for the surface” is used to describe competition at the implant surface between microbial adhesion and tissue integration (Gristina, 1987; Subbiahdoss et al., 2009; Caraca-Huber et al., 2012). The goal is to have tissue cells win the race against bacteria to prevent biofilm formation, which obstructs cellular functions and healthy tissue formation (Subbiahdoss et al., 2009). Additional complications caused by implant-associated infections include: BAIs are very difficult to manage and often require removal of the implant (Gorth et al., 2012); treatment costs are overwhelmingly expensive (Kurtz et al., 2008); and BAIs are very painful and debilitating to patients. For these reasons and as the number of implantations continue to rise, gaining a better understanding of how cells interact with biomaterial surfaces is critical. The sizes of the surface-fouling microorganisms are typically 1–2 μm (Graham and Cady, 2014), characteristically have less deformable membranes (compared to eukaryotic cells), and present distinctive structures (Anselme et al., 2010); therefore, using textured biomaterial surfaces may be an advantageous method to disrupt adhesion and mobility mechanisms of bacteria and limit biofilm formation.

In this article, increased cell spreading and random migration suggest better surface coverage and movement, which could help reduce the potential of BAIs. The cell aspect ratio and circularity data provide information about projected phenotype lineage commitment based on published literature investigating cell differentiation on surfaces similar to those presented (Matsuoka et al., 2013), and the differentiation data presented support these previous findings. This information will suggest effects of surface features presented to the cell based on common spinal fusion cage materials: textured rough titanium, smooth titanium, and PEEK. We hypothesize that the acid-etched endoskeleton surface will lead to cellular responses indicative of successful spinal implants by demonstrating cellular responses that suggest microtopography may be a possible key parameter in preventing biofilm formation. This research enhances the current understanding of cellular responses to biomaterials, detailed toward spinal fusion research, by giving insight into cellular responses as correlated with surface morphology of common biomaterials for spinal implants. In turn, this research could aid in improving the functional integrity and performance of spinal fusion devices.

## Materials and Methods

### Substrate Preparation

**Figure 1:**
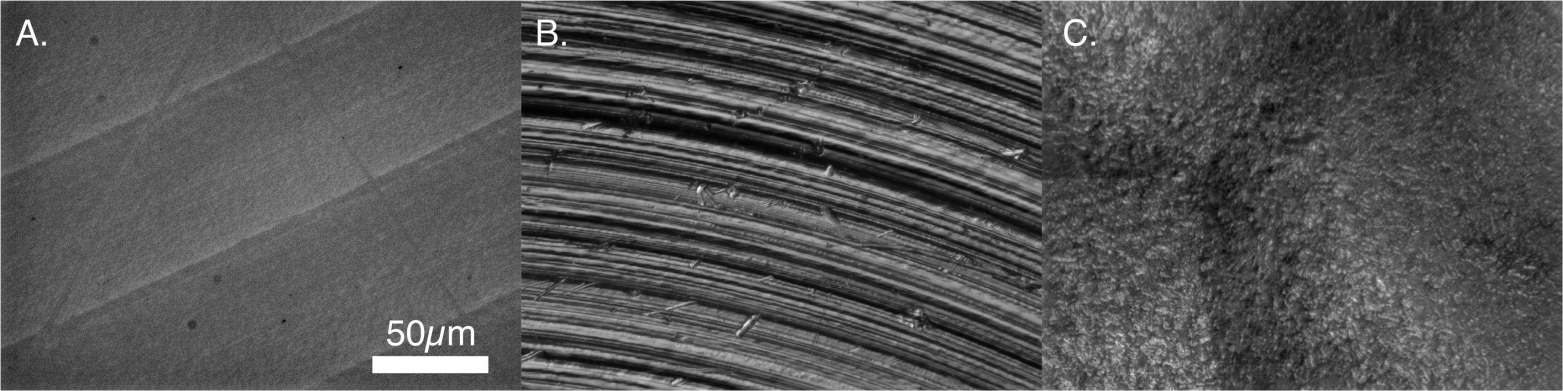
**Surface morphology of PEEK and titanium samples**. **(A)** PEEK, **(B)** smooth titanium, and **(C)** rough, acid-etched endoskeleton surface.

Substrates were 15-mm diameter disks machined from titanium alloy (Ti6Al4V ELI per ASTM F136) and PEEK (ASTM F2026) to create relatively smooth surfaces (Titan Spine, LLC, Mequon, WI, USA). To create the roughened surface texture, titanium disks were treated with the proprietary endoskeleton acid-etch process. Figure 1 presents the surface topographies of PEEK, smooth titanium, and acid-etched titanium (Matteson et al., 2015). All disks were sterilized by immersion in 70% ethanol for 30 min (Kummer et al., 2013; Vidal et al., 2013; Hirano et al., 2014) and rinsed with 1× phosphate-buffered saline (PBS) prior to use.

### Cell Culture

Human mesenchymal stem cells (MSCs) were obtained from Lonza and were grown to appropriate numbers in a humidified incubator at 37°C and 5% CO_2_, and then seeded onto surfaces at 1000 cells/cm^2^ for early morphology and early migration experiments. A lower seeding value, compared with the typical seeding value of 10000 cells/cm^2^, was chosen in order to be able to better characterize the morphology, spreading, and migration events. At the time of seeding, the MSCs were at passage 6. The MSCs were maintained in a basal growth media for all early morphology and early migration experiments. The basal growth media consisted of alpha modified MEM (Life Technologies, Carlsbad, CA, USA) supplemented with 10% fetal bovine serum (FBS, Atlanta Biologics, Atlanta, GA, USA) and 1% penicillin/streptomycin (Life Technologies, Carlsbad, CA, USA). The media was changed every 2 days during the culture period. For the differentiation studies, samples were seeded at 10000 cells/cm^2^ with a culture period of 10 days. Alpha-modified MEM basal growth media was switched to osteogenic media 12 h after seeding. Osteogenic media consisted of alpha modified MEM (Life Technologies, Carlsbad, CA, USA) supplemented with 10% fetal bovine serum (FBS, Atlanta Biologics, Atlanta, GA, USA), 1% penicillin/streptomycin (Life Technologies, Carlsbad, CA, USA), 100-nM dexamethasone (Sigma-Aldrich, St. Louis, MO, USA), 50 μg/mL ascorbic acid (Sigma-Aldrich, St. Louis, MO, USA), and 10-mM beta-glycerophosphate (Sigma-Aldrich, St. Louis, MO, USA).

### Early Morphology

Mesenchymal stem cells on surfaces used for early morphology were stained with the DiI derivative, DiR (Life Technologies, Carlsbad, CA, USA) to fluorescently label the cell membrane. The staining was carried out by incubating the MSCs in a solution of 0.5% DiR in basal media, from a stock DiR solution concentration of 1 mg/mL in ethanol, for 30 min in a humidified incubator at 37°C. The samples were maintained in the incubator and removed at 2, 6, and 24 h to acquire images. At each time point, a minimum of 31 cells was imaged for analysis for each sample. Due to the depth of the surface, *z*-stacks were acquired and processed using the extended depth of field plugin for ImageJ. The subsequent images were analyzed with MATLAB to create image masks and with CellProfiler to quantify cell morphology. A minimum *n* = 30 was used for each sample.

### Early Migration

Mesenchymal stem cells on surfaces used for early migration quantification were stained with a Qtracker 705 Cell Labeling kit (Life Technologies, Carlsbad, CA, USA) prior to being seeded. This allowed long-term evaluation of the cell centroid based on endocytosis of quantum dots. The quantum dot-loaded cells were imaged every 10 min for 12 h. Similar to the cell morphology, *z*-stacks were acquired at each time point and processed with the extended depth of field plugin for ImageJ. CellProfiler was used to quantify the migration velocities and directions. A minimum *n* = 6 was used for each sample.

### Differentiation Markers

At 10 days, samples were lysed with 200-μL radioimmunoprecipitation assay buffer. The lysates were used to quantify alkaline phosphatase (ALP), osterix (OSX) (SP7) transcription factor levels, and double-stranded DNA. General protocols for the immunodetection ALP substrate kit (Bio-Rad, Hercules, CA, USA), Quant-iT™ Picogreen^®^ dsDNA reagent kit (Invitrogen, Molecular Probes), and Human SP7/Osterix ELISA Kit (LifeSpan BioSciences, Inc., Seattle, WA, USA) were followed as written. Sample volumes run in the assays were 5 μL.

### Late Nuclear Morphology and Immunostaining

At 24 h and 7 days, samples were removed and fixed for immunostaining in addition to analysis of nuclear morphology. The samples to be stained for imaging at the 24-h time point were quickly washed with cold PBS and then fixed with 3.7% paraformaldehyde for 15 min followed by permeabilization in 0.1% Triton X-100 in 2% bovine serum albumin for 1 h. The cells were incubated with a mouse monoclonal anti-vinculin antibody (Sigma-Aldrich, St. Louis, MO, USA) at 1:400 in the permeabilization buffer for 1 h at room temperature. The samples were washed with PBS three times and then incubated in phalloidin conjugated to Atto 490LS (Sigma-Aldrich, St. Louis, MO, USA) at 1:1000, DAPI at 1:1000, and Dylight 488 anti-mouse secondary antibody (Life Technologies, Carlsbad, CA, USA) at 1:200 for 1 h at room temperature. Finally, the samples were washed three times with PBS and imaged. The samples imaged after 7 days were prepared as above; however, the vinculin primary and 488 secondary were omitted since vinculin was not needed to determine morphology. Nuclear morphology was quantified with CellProfiler.

### Statistics

One-way ANOVAs with Tukey *post hoc* tests were used to determine significant differences for morphology features measured, migration velocity and migration directionality, and nuclear area and axial rotation. A *χ*^2^ was used to evaluate the alignment of nuclei in each sample relative to a predicted random distribution.

## Results

### Early Morphology

**Figure 2:**
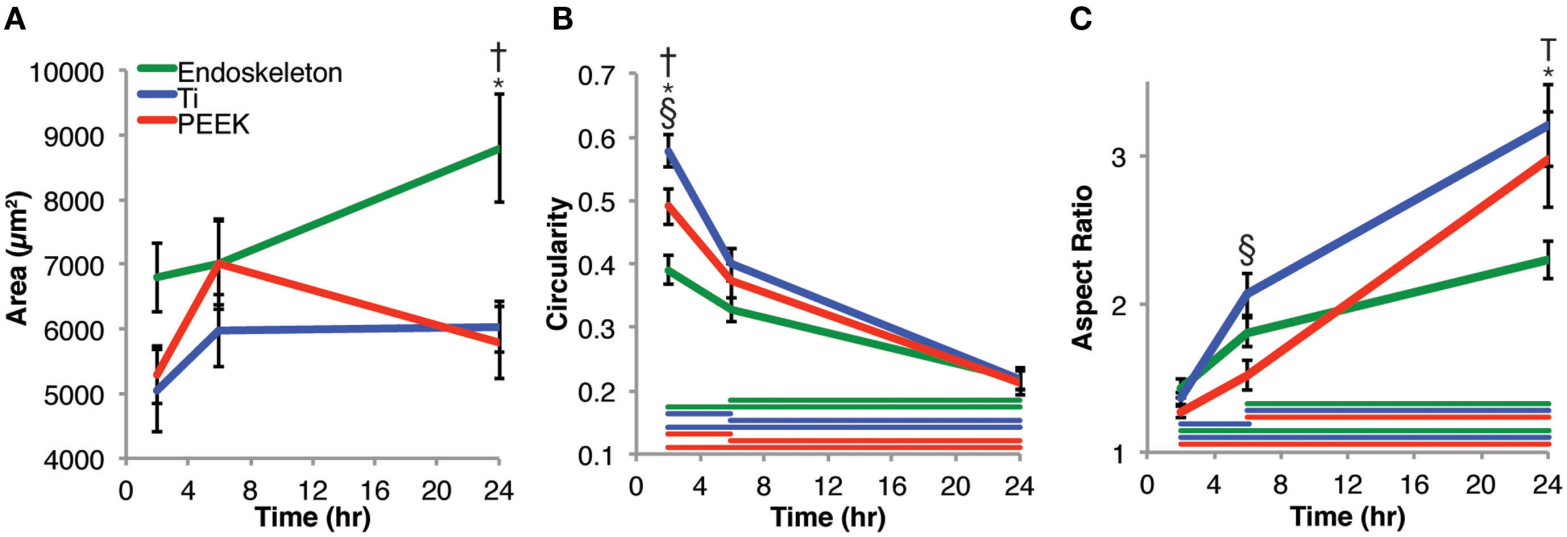
**Morphological changes of mesenchymal stem cells analyzed at 2, 6, and 24 h post-seeding**. **(A)** Area, **(B)** circularity, and **(C)** aspect ratio measurements were taken. The results indicate that stem cells on the acid-etched endoskeleton surface spread the most over 24 h. The circularity of the three surfaces began dissimilar, but converged at 24 h. The aspect ratio of stem cells initially began close to 1, but over 24 h, the smooth surfaces, Ti and PEEK, increased significantly higher than the rough, acid-etched endoskeleton surface. Taken together, the aspect ratio and circularity indicate that stem cells on smooth surfaces move toward a spindle or fibroblastic morphology, whereas those on the rough, acid-etched endoskeleton surface moved toward a stellate or star-like morphology. Within a single time point, * indicates significance, *p* < 0.05 between acid-etched Ti and PEEK, ^†^ indicates significance between acid-etched Ti and Ti, and ^§^ indicates significance between PEEK and Ti. Color-coded bars demonstrate significance between time points for a single surface.

Mesenchymal stem cell morphology was examined both quantitatively from 2 to 24 h and qualitatively at 24 h. Figure 2 presents the quantitative cell morphology results on smooth PEEK, smooth Ti, and acid-etched endoskeleton surfaces. Figure 2A presents the areas of MSCs on each surface and shows an increasing trend for MSCs on the acid-etched endoskeleton surface: 6801 ± 533 μm^2^ at 2 h, 7016 ± 647 μm^2^ at 6 h, and ending with 8795 ± 841 μm^2^ at 24 h. Similarly, the spreading area of MSCs on the smooth Ti increased at each time point: 5047 ± 634 μm^2^ at 2 h, 5971 ± 562 μm^2^ at 6 h, and 6041 ± 396 μm^2^ at 24 h. However, the smooth PEEK was the only surface to demonstrate a maximal value followed by a decrease: 5292 ± 442 μm^2^ at 2 h, 7008 ± 702 μm^2^ at 6 h, and falling back to 5791 ± 565 μm^2^ at 24 h. Only the acid-etched endoskeleton surface demonstrated significance with respect to area, demonstrating more spreading area per cell at 24 h when compared to the other surfaces at 24 h in addition to the earlier time points on the acid-etched surface. Next, the circularity of MSCs on the surfaces was analyzed. Circularity was defined as

$$\text{Circularity}=\frac{4\pi A}{P^{2}}$$

**Figure 3:**
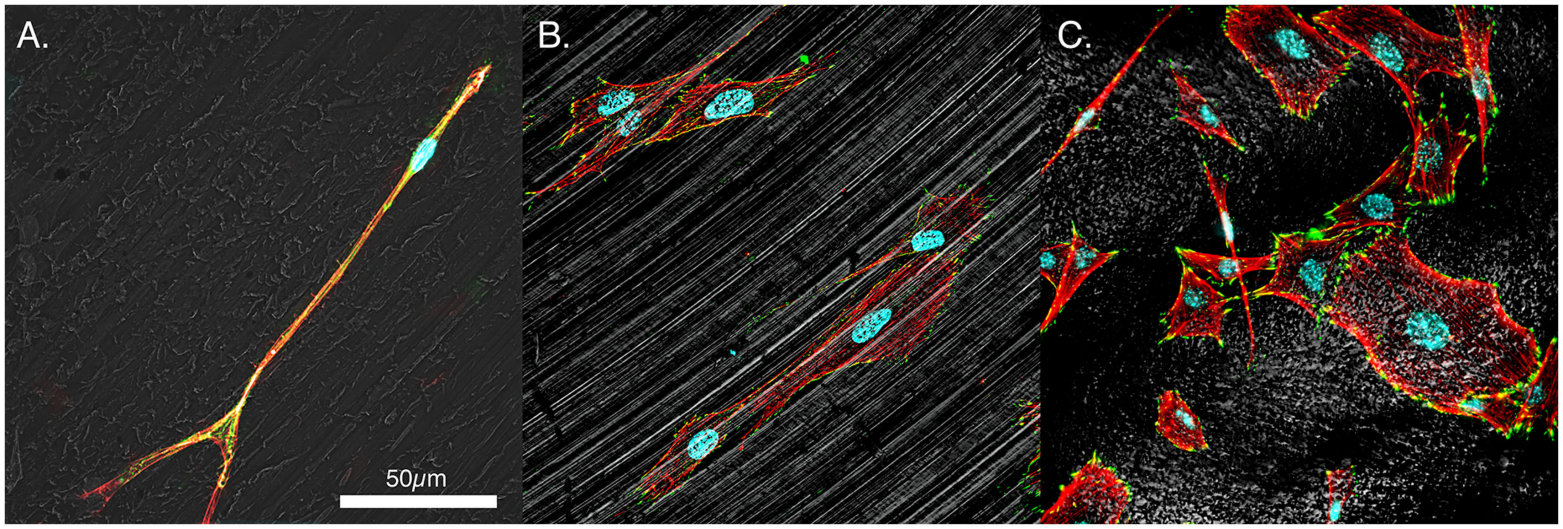
**Representative morphologies of MSCs**. **(A)** PEEK, **(B)** smooth titanium, and **(C)** rough, acid-etched endoskeleton surface, at 24 h. Immunofluorescence was carried out to examine the focal adhesion protein vinculin (green), the actin cytoskeleton (red), and the cell nuclei (blue). Additionally, a gray scale depiction of the surface was obtained with reflected DIC. The results demonstrated the trends observed in Figure 2 with cells on the smooth surfaces moving toward an elongated spindle-shaped morphology, whereas the cells on the rough surface demonstrated a range of morphologies from spindle-shaped cells to cuboidal and stellate-shaped cells. In particular, the cuboidal and stellate cells in C. are representative of morphologies expected of osteoblastic differentiation. Scale bar indicating 50 μm applies to **(A–C)**.

Mesenchymal stem cells on all three surfaces demonstrated a significant decrease in the circularity of the cells at each time point. Smooth Ti surfaces demonstrated the highest circularity at 2 h, whereas the acid-etched endoskeleton surfaces demonstrated the lowest circularity at 2 h. The final shape factor analyzed was aspect ratio. All three surfaces demonstrated an increasing aspect ratio at each subsequent time point. The two smooth surfaces reached a final aspect ratio at 24 h of approximately 3, whereas the acid-etched endoskeleton surface reached a final aspect ratio of approximately 2. At 24 h, samples were stained to qualitatively examine morphology. Figure 1 illustrates the surface topography of the samples; reflected DIC was used to obtain the images. Figure 3 depicts the results from the qualitative staining, which are stained for phalloidin (red), the adhesion protein vinculin (green), and nuclear DNA (blue). In each image, a reflected DIC image of the surface is overlaid with the fluorescence channels in gray. In Figures 3A,B, it is evident that MSCs on the smooth surfaces demonstrate an elongated spindle-like morphology. In contrast to Figures 3A–C, demonstrates MSCs with cuboidal and stellate (star-shaped) morphologies on the acid-etched endoskeleton surface.

### Early Migration

**Figure 4:**
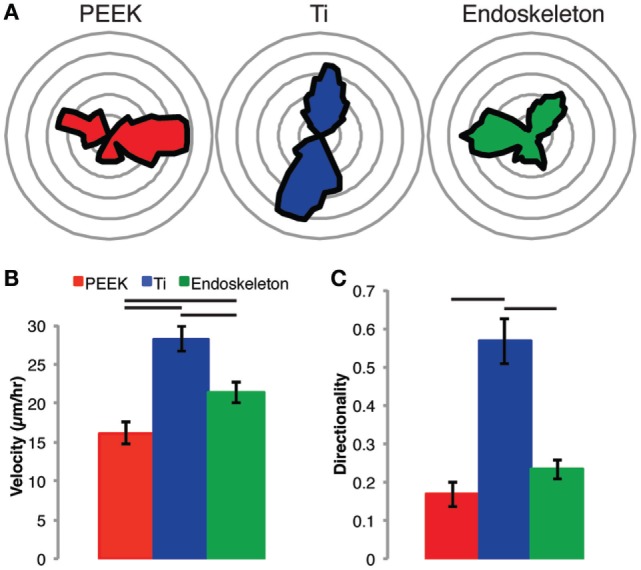
**Stem cell migration on each surface was assessed from 6 to 18 h post-seeding**. The results demonstrate random migration on the PEEK and acid-etched endoskeleton surfaces indicated by the rose plots in **(A)**, a histogram of the angle of migration for each cell monitored in **(B)**, and the graph of directionality in **(C)**, which demonstrates significance between PEEK and acid-etched endoskeleton surfaces when compared to the smooth titanium surface. Furthermore, the non-random migration on smooth titanium followed the grooves created by milling the surface, and this non-random migration resulted in an expected velocity increase, which was significantly higher than both the PEEK and acid-etched endoskeleton surfaces. Between the two surfaces demonstrating random migration, the MSCs on the acid-etched endoskeleton surface demonstrated a significantly higher velocity than those on PEEK. Significance, *p* < 0.05, is demonstrated by bars between groups in **(B,C)**.

In addition to the morphology shift on each of the three surfaces, the rate and direction of migration were assayed beginning at 6 h post-seeding and continuing for 12 h. Figure 4 depicts the quantitative migration data. In Figure 4A, rose plots (circular histograms) are provided to demonstrate the direction of travel. The MSCs on both smooth surfaces (e.g., PEEK and Ti) demonstrate migration along predominantly one axis. In contrast, the MSCs on the acid-etched endoskeleton surface demonstrate migration in multiple directions. The velocity on the samples is depicted in Figure 4B. The highest average velocity was found in MSCs on the smooth Ti, 28.24 ± 1.62 μm/h, followed by the acid-etched endoskeleton, 21.39 ± 1.38 μm/h, and the lowest average velocity was observed on smooth PEEK, 16.16 ± 1.46 μm/h. Velocities on each surface were significantly different than all other surfaces. Finally, the directionality of the cells on each surface was measured. Directionality was defined as

$$\text{Directionality}=\frac{\text{end-to-end distance}}{\text{total distance}}$$

The directionality was again highest on the smooth Ti, 0.57 ± 0.06, followed by the acid-etched endoskeleton, 0.23 ± 0.03, and the lowest directionality was observed on smooth PEEK, 0.17 ± 0.03. The smooth Ti was significantly higher as compared to both the smooth PEEK and the acid-etched endoskeleton surface.

### Differentiation Markers

**Figure 5:**
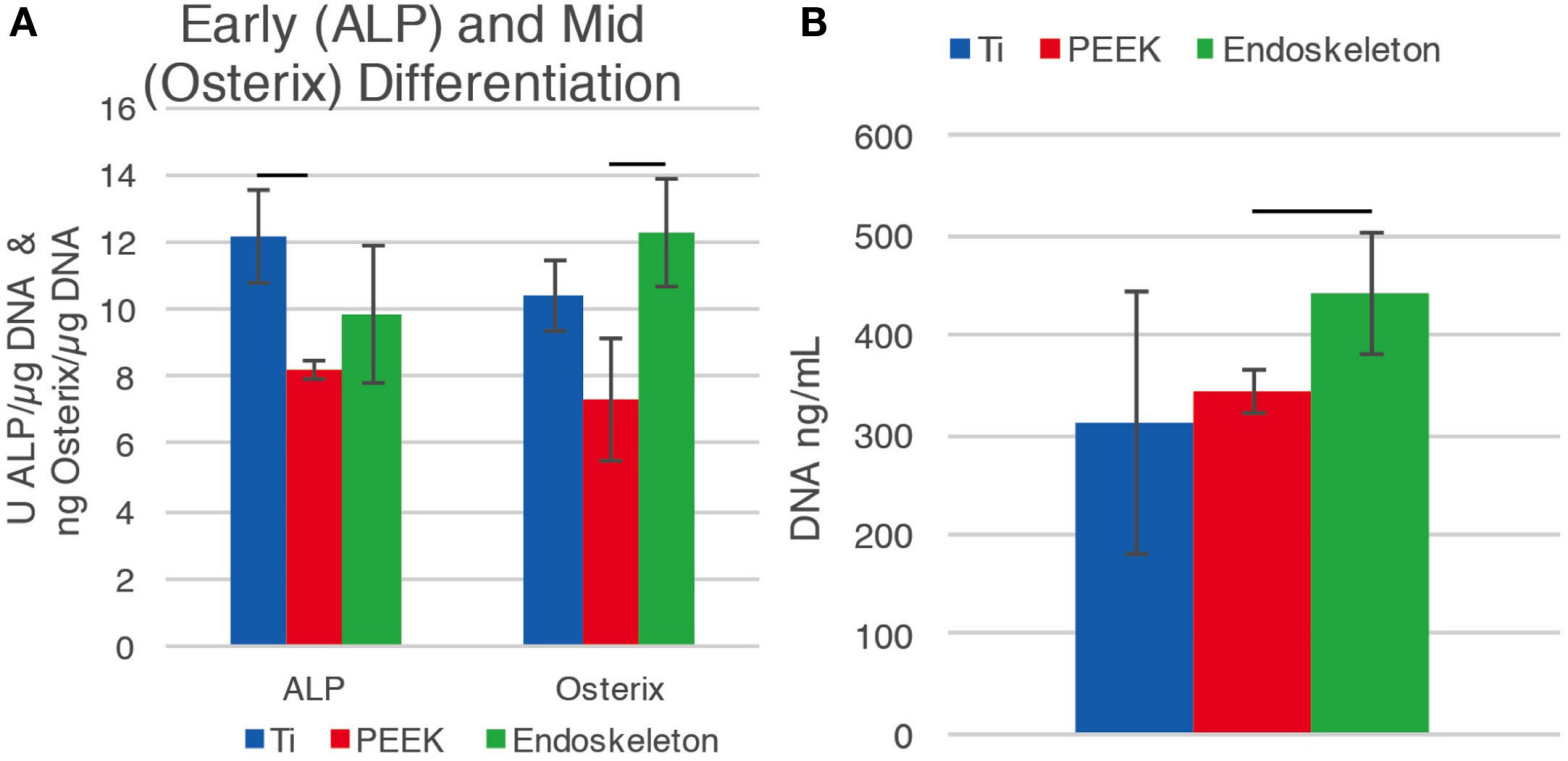
**Early differentiation marker alkaline phosphatase (ALP) and osterix (OSX), a transcription factor significant for osteoblast differentiation, were normalized to dsDNA**. **(A)** Early differentiation marker, ALP, is increased on the smooth Ti surface, while the mid-differentiation marker OSX increased on the acid-etched endoskeleton surface. The PEEK surface fell short for both ALP and OSX. This suggests that hMSC differentiation is moving toward bone formation for the Ti surfaces. **(B)** Seeding densities for all samples were equal. The dsDNA value for the acid-etched endoskeleton surface is the highest amongst the surfaces, which suggests that there is improved cell attachment and/or proliferation for the acid-etched endoskeleton surface compared to the smooth Ti and PEEK substrates.

The early osteogenic differentiation marker ALP and mid-differentiation marker OSX were investigated after 10 days on the three surfaces. dsDNA was quantified to normalize the ALP and OSX values. Figure 5A demonstrates that early maker, ALP, increased on the smooth Ti surface relative to PEEK, while the mid-marker OSX increased on the acid-etched endoskeleton surface. There was a significant difference between the ALP values for smooth Ti, 12.16 ± 1.37 U/μg, compared to PEEK, 8.19 ± 0.28 U/μg. The OSX value for the acid-etched endoskeleton surface was significantly higher than PEEK, 12.28 ± 1.59 and 7.31 ± 1.79 ng/μg, respectively. PEEK had decreased values for both ALP and OSX. Figure 5B shows a general increase of dsDNA from smooth Ti to PEEK to the acid-etched endoskeleton surface, 310.28 ± 131.90 to 342.41 ± 21.14 to 441.63 ± 60.45 ng/mL, with the acid-etched endoskeleton surface exhibiting the highest dsDNA value.

### Late Nuclear Morphology

**Figure 6:**
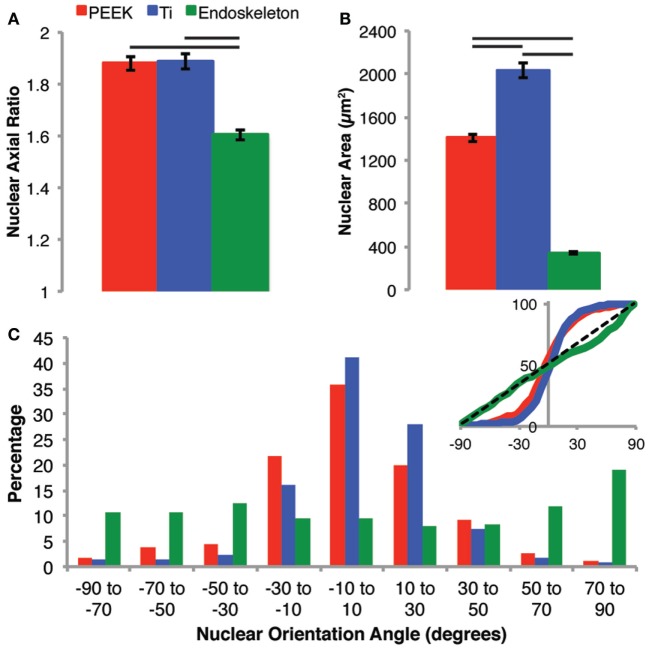
**Nuclear morphology was examined to assess the general cell morphology after 7 days when the populations were confluent and cell borders were difficult to identify**. The nuclear morphology on PEEK and Ti surfaces were very similar in regards to axial ratio **(A)**, whereas the nuclei on the rough acid-etched endoskeleton surface had a significantly lower axial ratio than either the PEEK or smooth Ti surface indicating more circular nuclei on the acid-etched endoskeleton surface. The nuclear area **(B)** followed a similar trend to axial ratio with the smooth surfaces demonstrating significantly more nuclear area than the rough acid-etched endoskeleton surface. Finally, the orientation of nuclei **(C)** was assessed establishing 0° as the average orientation direction. The inset provides a plot of the cumulative distribution and clearly demonstrates that PEEK and smooth Ti surfaces were different than the rough, acid-etched endoskeleton surface. Nuclei on PEEK and smooth Ti were grouped very close to 0° indicating that most cells presented an elongated nucleus in the same direction; however, on the acid-etched endoskeleton surface, the nuclei were randomly oriented with only one range, 70–90°, demonstrating a slight increase. The black dotted line in the inset of **(C)** provides the expected cumulative distribution for random orientation; *p* values were calculated for each of the three samples with a *χ*^2^ test and yielded *p* values of 10^−10^, 10^−19^, and 1.0 for PEEK, Ti, and acid-etched endoskeleton, respectively. Taken together, these results indicated that the aligned spindle morphology observed early on the PEEK and smooth Ti surfaces persists when the stem cells are confluent, and likewise, the random cuboidal/stellate morphology on the acid-etched endoskeleton surface also persists to the confluent cell layer observed after 7 days.

After 7 days, the nuclei morphologies were analyzed as a predictor of cell morphology. The cell morphology could not be assessed directly at 7 days due to the cells being confluent; however, nuclear morphology has previously demonstrated a correlation over the general cell morphology (Maniotis et al., 1997; Li et al., 2014; Ramdas and Shivashankar, 2015). First, we examined the axial ratio and nuclear area on all three surfaces, shown in Figure 6A. The axial ratios were similar on the PEEK and smooth Ti, 1.88 ± 0.03 and 1.89 ± 0.03, respectively. The axial ratio for nuclei on the acid-etched endoskeleton surface, 1.60 ± 0.02, was significantly lower than both of the smooth surfaces. The acid-etched endoskeleton surface also demonstrated the lowest nuclear area, 340.5 ± 9.3 μm^2^, as compared to the PEEK and smooth Ti surfaces, 1406.4 ± 31.4 and 2037.6 ± 70.8 μm^2^. All nuclear areas were significantly different from all others, illustrated in Figure 6B. Finally, the alignment of the nuclei was quantified by examining the angle of the long axis of each nucleus. This data is presented in a histogram in Figure 6C. Additionally, the inset of Figure 6C provides a cumulative distribution plot of the histogram data with a dotted line corresponding to a random distribution. A *X*^2^ (chi-squared) test demonstrated that both the PEEK and smooth Ti had nuclei aligned in a single direction, which was significantly different than an expected random distribution. In contrast, the nuclei on the acid-etched endoskeleton surface were not significantly different from an expected random distribution.

**Figure 7:**
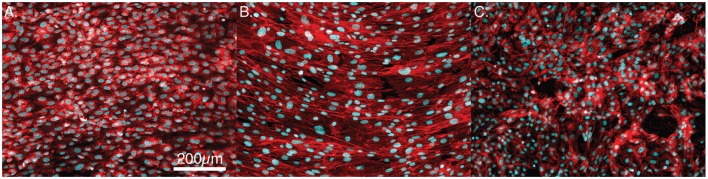
**Representative images of confluent cells stained for actin (red) and the cell nuclei (blue)**. **(A)** PEEK, **(B)** smooth titanium, and **(C)** rough, acid-etched endoskeleton surface, after 7-day culture. The cells on PEEK and smooth titanium demonstrate an elongated morphology in a uniform direction, whereas cells on the acid-etched endoskeleton surface demonstrate a branched random morphology. Scale bar indicating 200 μm applies to **(A–C)**.

The quantitative data above are represented by the immunofluorescence images presented in Figures 7A–C. Figures 7A,B show confluent layers of MSCs on the PEEK and smooth Ti surfaces. In each, it is clear that the nuclei are elongated and are primarily organized along a single axis. In contrast, the acid-etched endoskeleton surface presents a more random distribution of MSC morphologies.

## Discussion

The advantages of the acid-etched endoskeleton surface in promoting osteoblast differentiation are well established in the literature (Gittens et al., 2012; Olivares-Navarrete et al., 2012, 2013). The goals of the present study were to examine the early responses of MSCs to each of the surfaces, and identify if the early response was predictive of the known long-term osteoblastic differentiation and establishment of MSC migration and morphology. The project objective was to demonstrate data characterizing the events preceding phenotype development on rough titanium since material and surface characterization and cell differentiation data with respect to rough versus smooth surfaces have been well studied. A key feature of successful implants is winning the “race for the surface” (Gristina et al., 1989). This can be defined by examining key features: rate of initial cell adhesion and spreading and the rate of random cell migration. Random cell migration is significant in generating the uniform population distribution that will lead to uniform coverage of the surface by the MSCs (Gail and Boone, 1970). Examination of the data from the cell area measurements clearly reveals that MSCs on the acid-etched endoskeleton surface spread at a higher rate than on either of the smooth surfaces. This was demonstrated by a 40% increase in spreading area per cell by 2 h for MSCs on the acid-etched endoskeleton surface, which increased to nearly 50% more spreading area per cell by 24 h. A 50% increase in the cell spreading area dramatically reduces the time required for the surface to be covered by MSCs. Furthermore, the shape of these spreading cells revealed interesting trends. Initially, all MSCs were circular, or nearly circular, as demonstrated by high circularity values and low aspect ratios. As time progressed, MSCs on the smooth surfaces moved toward spindle-shaped morphologies. These elongated and aligned spindle-shaped cells are typical morphology of fibroblastic tissue (Dalby et al., 2007). This is evident from the combination of a high aspect ratio, 3:1 at 24 h, coupled with low circularity. Together, these two characteristics suggest long slender cells that are further supported by the immunostaining images at 24 h presented in Figure 3. In contrast, MSCs on the acid-etched endoskeleton surface demonstrated more stellate or cuboidal morphologies at 24 h, indicated by lower aspect ratios and similar circularities. The similar circularity indicates that MSCs on the acid-etched endoskeleton surface have a high perimeter:area ratio, but the lower circularity indicates that the long axis and short axis of the cell are not dramatically different. Together, these two properties lead to the conclusion that MSCs on the acid-etched endoskeleton surface have multiple processes and are stellate in morphology. Figure 3 demonstrates MSCs on the acid-etched endoskeleton surface showing varied morphologies from stellate to spindle-shaped. The cuboidal and stellate morphologies observed on the acid-etched endoskeleton surface are expected for cells undergoing osteogenic differentiation (Dalby et al., 2007; Hong et al., 2010). Furthermore, the differentiation data illustrated in Figure 5A support that the cells were indeed moving toward the osteogenic lineage as indicated by increased levels of ALP and OSX compared to PEEK (Pinzone et al., 2016). The increased ALP on smooth Ti and increased OSX on acid-etched endoskeleton surface together suggest that osteogenic differentiation was dramatically enhanced on the acid-etched endoskeleton surface, with smooth Ti being moderately enhanced relative to PEEK. The dsDNA data in Figure 5B also suggest improved cell attachment and/or proliferation for the acid-etched endoskeleton surface based on the increased dsDNA value for that surface and the fact that initial seeding densities across the samples were equal.

The examination of migration further supported the acid-etched endoskeleton surface. MSCs on both of the titanium surfaces were faster than those on PEEK. The MSCs on the acid-etched endoskeleton surface did not present the highest velocity; however, they did present both a moderately high velocity coupled with a low directionality. The low directionality and histogram of migration vectors indicate the MSCs on the acid-etched endoskeleton surface were moving in a random way. In contrast, MSCs on the smooth titanium exploited contact guidance generated by machining the titanium. This contact guidance resulted in high directionality with migration occurring primarily on one axis. The MSCs on PEEK presented random migration; however, the MSCs velocities were significantly lower than either of the two titanium surfaces.

Together, the morphology and migration data support the acid-etched endoskeleton surface as the best option for randomly and uniformly distributing the cell population in addition to covering the available area when compared to either of the smooth surfaces. Furthermore, the acid-etched endoskeleton surface promoted bone differentiation markers after 10 days and stellate and cuboidal cell morphologies as early as 24 h, which are the expected morphologies of MSCs undergoing osteogenic differentiation. Finally, the long-term examination of nuclear morphology at 7 days indicates that the morphologies observed at 24 h persisted on the surfaces. Both of the smooth surfaces presented spindle-shaped cells, and at 7 days, the nuclei were elongated along a uniform axis. Therefore, it may be concluded that the cells are still spindle-shaped when confluent. In contrast, MSCs on the acid-etched endoskeleton surface did not demonstrate preferential nuclei alignment, which indicates that MSCs on the acid-etched endoskeleton surface maintained a cuboidal or stellate morphology at 7 days. In closing, the unique rough surface presented by the acid-etched endoskeleton surfaces promotes morphology and migration behavior consistent with successful implants.

## Author Contributions

BB contributed to the acquisition of data, drafting of manuscript, and critical revision. TR and CP contributed to the acquisition and analysis of the data. JB contributed through study design, analysis and interpretation of data, and drafting of the manuscript. All authors critically reviewed the work, approved the final version to be published, and agree to be accountable for the work.

## Conflict of Interest Statement

The authors declare that the research was partially funded by Titan Spine, LLC.

## References

[B1] Abernathie D. L. Pfeiffer F. M. (2011). Spinal Fusion Cage, Method of Design, and Method of Use. US 8,057,548 B2.

[B2] Anselme K. Bigerelle M. (2005). Topography effects of pure titanium substrates on human osteoblast long-term adhesion. Acta Biomater. 1, 211–222. 10.1016/j.actbio.2004.11.009 16701798

[B3] Anselme K. Davidson P. Popa A. M. Giazzon M. Liley M. Ploux L. (2010). The interaction of cells and bacteria with surfaces structured at the nanometre scale. Acta Biomater. 6, 3824–3846. 10.1016/j.actbio.2010.04.001 20371386

[B4] Bächle M. Kohal R. J. (2004). A systematic review of the influence of different titanium surfaces on proliferation, differentiation and protein synthesis of osteoblast-like MG63 cells. Clin. Oral Implants Res. 15, 683–692. 10.1111/j.1600-0501.2004.01054.x 15533129

[B5] Cabraja M. Oezdemir S. Koeppen D. Kroppenstedt S. (2012). Anterior cervical discectomy and fusion: comparison of titanium and polyetheretherketone cages. BMC Musculoskelet. Disord. 13:172. 10.1186/1471-2474-13-172 22978810 PMC3493386

[B6] Caraca-Huber D. C. Fille M. Hausdorfer J. Pfaller K. Nogler M. (2012). Evaluation of MBEC^TM^-HTP biofilm model for studies of implant associated infections. J. Orthop. Res. 30, 1176–1180. 10.1002/jor.22065 22228044

[B7] Dalby M. J. Gadegaard N. Tare R. Andar A. Riehle M. O. Herzyk P. (2007). The control of human mesenchymal cell differentiation using nanoscale symmetry and disorder. Nat. Mater. 6, 997–1003. 10.1038/nmat2013 17891143

[B8] Deligianni D. D. Katsala N. Ladas S. Sotiropoulou D. Amedee J. Missirlis Y. F. (2001). Effect of surface roughness of the titanium allow Ti-6Al-4V on human bone marrow cell response and on protein adsorption. Biomaterials 22, 1241–1251. 10.1016/S0142-9612(00)00274-X 11336296

[B9] Gail M. H. Boone C. W. (1970). The locomotion of mouse fibroblasts in tissue culture. Biophys. J. 10, 980–993. 10.1016/S0006-3495(70)86347-0 5531614 PMC1367974

[B10] Gittens R. A. Olivares-Navarrete R. McLachlan T. Cai Y. Hyzy S. L. Schneider J. M. (2012). Differential responses of osteoblast lineage cells to nanotopographically-modified, microroughened titanium-aluminum-vanadium alloy surfaces. Biomaterials 33, 8986–8994. 10.1016/j.biomaterials.2012.08.059 22989383 PMC3618458

[B11] Gorth D. J. Puckett S. Ercan B. Webster T. J. Rahaman M. Bal B. S. (2012). Decreased bacteria activity on Si3N4 surfaces compared with PEEK or titanium. Int. J. Nanomed. 7, 4829–4840. 10.2147/IJN.S35190 PMC3439860 22973102

[B12] Graham M. V. Cady N. C. (2014). Nano and microscale topographies for the prevention of bacterial surface fouling. Coatings 4, 37–59. 10.3390/coatings4010037

[B13] Gristina A. (1987). Biomaterial-centered infection: microbial adhesion versus tissue integration. Science 237, 1588–1595. 10.1126/science.3629258 3629258

[B14] Gristina A. Naylor P. Myrvik Q. (1989). Infections from biomaterials and implants: a race for the surface. Med. Prog. Technol. 14, 205–224. 2978593

[B15] Higgins A. M. Banik B. L. Brown J. L. (2015). Geometry sensing through POR1 regulates Rac1 activity controlling early osteoblast differentiation in response to nanofiber diameter. Integr. Biol. 7, 229–236. 10.1039/c4ib00225c 25539497 PMC4323730

[B16] Hirano M. Kozuka T. Asano Y. Kakuchi Y. Arai H. Ohtsu N. (2014). Effect of sterilization and water rinsing on cell adhesion to titanium surfaces. Appl. Surf. Sci. 311, 498–502. 10.1016/j.apsusc.2014.05.096

[B17] Hong D. Chen H.-X. Yu H.-Q. Liang Y. Wang C. Lian Q.-Q. (2010). Morphological and proteomic analysis of early stage of osteoblast differentiation in osteoblastic progenitor cells. Exp. Cell Res. 316, 2291–2300. 10.1016/j.yexcr.2010.05.011 20483354 PMC4580249

[B18] Kummer K. M. Taylor E. N. Durmas N. G. Tarquinio K. M. Ercan B. Webster T. J. (2013). Effects of different sterilization techniques and varying anodized TiO_2_ nanotube dimensions on bacteria growth. J. Biomed. Mater. Res. B Appl. Biomater. 101, 677–688. 10.1002/jbm.b.32870 23359494

[B19] Kurtz S. M. Lau E. Schmier J. Ong K. L. Zhao K. Parvizi J. (2008). Infection burden for hip and knee arthroplasty in the United States. J. Arthroplasty 23, 984–991. 10.1016/j.arth.2007.10.017 18534466

[B20] Li Q. Kumar A. Makhija E. Shivashankar G. V. (2014). The regulation of dynamic mechanical coupling between actin cytoskeleton and nucleus by matrix geometry. Biomaterials 35, 961–969. 10.1016/j.biomaterials.2013.10.037 24183171

[B21] Maniotis A. J. Chen C. S. Ingber D. E. (1997). Demonstration of mechanical connections between integrins, cytoskeletal filaments, and nucleoplasm that stabilize nuclear structure. Proc. Natl. Acad. Sci. U.S.A. 94, 849–854. 10.1073/pnas.94.3.849 9023345 PMC19602

[B22] Matsuoka F. Takeuchi I. Agata H. Kagami H. Shiono H. Kiyota Y. (2013). Morphology-based prediction of osteogenic differentiation potential of human mesenchymal stem cells. PLoS ONE 8:e55082. 10.1371/journal.pone.0055082 23437049 PMC3578868

[B23] Matteson J. L. Greenspan D. C. Tighe T. B. Gilfoy N. Stapleton J. J. (2015). Assessing the hierarchical structure of titanium implant surfaces. J. Biomed. Mater. Res. Part B. 10.1002/jbm.b.33462 26034005

[B24] Nouh M. R. (2012). Spinal fusion-hardware construct: basic concepts and imaging review. World J. Radiol. 4, 193–207. 10.4329/wjr.v4.i5.193 22761979 PMC3386531

[B25] Obrigkeit D. D. Koenen J. Schumann D. O. A. Smit L. (2012). Spinal Fusion Cage. US 2012/0046750 A1.

[B26] Olivares-Navarrete R. Gittens R. A. Schneider J. M. Hyzy S. L. Haithcock D. A. Ullrich P. F. (2012). Osteoblasts exhibit a more differentiated phenotype and increased bone morphogenetic protein production on titanium alloy substrates than on poly-ether-ether ketone. Spine J. 12, 265–272. 10.1016/j.spinee.2012.02.002 22424980 PMC3618467

[B27] Olivares-Navarrete R. Hyzy S. Hutton D. Erdman C. Wieland M. Boyan B. D. (2010). Direct and indirect effects of microstructured titanium substrates on the induction of mesenchymal stem cell differentiation towards the osteoblast lineage. Biomaterials 31, 1–15. 10.1016/j.biomaterials.2009.12.029 20053436 PMC2821717

[B28] Olivares-Navarrete R. Hyzy S. L. Gittens R. A. I Schneider J. M. Haithcock D. A. Ullrich P. F. (2013). Rough titanium alloys regulate osteoblast production of angiogenic factors. Spine J. 13, 1563–1570. 10.1016/j.spinee.2013.03.047 23684238 PMC3785549

[B29] Ozdemir T. Xu L.-C. Siedlecki C. Brown J. L. (2013). Substrate curvature sensing through Myosin IIa upregulates early osteogenesis. Integr. Biol. 5, 1407–1416. 10.1039/c3ib40068a 24104522

[B30] Pinzone J. J. Hall B. M. Thudi N. K. Vonau M. Qiang Y. Rosol T. J. (2016). The role of Dickkopf-1 in bone development, homeostasis, and disease. Blood 113, 517–526. 10.1182/blood-2008-03-145169 18687985 PMC2628360

[B31] Ramdas N. M. Shivashankar G. V. (2015). Cytoskeletal control of nuclear morphology and chromatin organization. J. Mol. Biol. 427, 695–706. 10.1016/j.jmb.2014.09.008 25281900

[B32] Rihn J. A. Patel R. Makda J. Hong J. Anderson D. G. Vaccaro A. R. (2009). Complications associated with single-level transforaminal lumbar interbody fusion. Spine J. 9, 623–629. 10.1016/j.spinee.2009.04.004 19482519

[B33] Subbiahdoss G. Kuijer R. Grijpma D. W. van der Mei H. C. Busscher H. J. (2009). Microbial biofilm growth versus tissue integration: “the race for the surface” experimentally studied. Acta Biomater. 5, 1399–1404. 10.1016/j.actbio.2008.12.011 19158003

[B34] Vidal G. Blanchi T. Mieszawska A. J. Calabrese R. Rossi C. Vigneron P. (2013). Enhanced cellular adhesion on titanium by silk functionalized with titanium binding and RGD peptides. Acta Biomater. 9, 4935–4943. 10.1016/j.actbio.2012.09.003 22975628 PMC3508072

[B35] Williams A. L. Gornet M. F. Burkus J. K. (2005). CT evaluation of lumbar interbody fusion: current concepts. Am. J. Neuroradiol. 26, 2057–2066. 16155160 PMC8148852

[B36] Zhao C. Cao P. Ji W. Han P. Zhang J. Zhang F. (2011). Hierarchical titanium surface textures affect osteoblastic functions. J. Biomed. Mater. Res. A 99, 666–675. 10.1002/jbm.a.33239 21972107

